# Editorial: Plant Glutathione Transferases: Diverse, Multi-Tasking Enzymes With Yet-to-Be Discovered Functions

**DOI:** 10.3389/fpls.2019.01304

**Published:** 2019-10-18

**Authors:** Jolán Csiszár, Arnaud Hecker, Nikolaos E. Labrou, Peter Schröder, Dean E. Riechers

**Affiliations:** ^1^Department of Plant Biology, Faculty of Science and Informatics, University of Szeged, Szeged, Hungary; ^2^Interactions Arbres-Microorganismes, Institut National de la Recherche Agronomique, Université de Lorraine, Nancy, France; ^3^Laboratory of Enzyme Technology, Department of Biotechnology, School of Applied Biology and Biotechnology, Agricultural University of Athens, Athens, Greece; ^4^Research Unit for Comparative Microbiome Analyses, Department of Environmental Sciences, Helmholtz Zentrum München, German Research Center for Environmental Health (GmbH), Neuherberg, Germany; ^5^Department of Crop Sciences, University of Illinois at Urbana-Champaign, Urbana, IL, United States

**Keywords:** glutathione transferases, catalysis, detoxification, glutathionylation, ligand binding, redox state, gene regulation, secondary metabolism

Plant genomes contain dozens of *GSTs* (Chi et al., 2011) encoding subunits that can form homodimers or heterodimers, leading to enormous diversity within GST protein families (Labrou et al., 2015). From its inception, plant GST research has successfully focused on investigating catalytic reactions with xenobiotic substrates (Cummins et al., 2011). By contrast, relatively few plant GST studies have successfully identified natural roles for this versatile multifunctional enzyme class, and despite few exceptions (Mueller et al., 2000; Bjarnholt et al., 2018), major breakthroughs have eluded researchers investigating their endogenous substrates and functions. With recent progress in molecular-genetics, physiology, and biochemistry, coupled with greatly increased sensitivity of mass spectrometry, it is timely to revisit potential candidates for natural GST substrates regarding catalysis, ligand binding, and transport roles, as well as summarize recent reports on xenobiotic detoxification and gene regulation mechanisms.

Cellular membrane lipids may become oxidized in plants growing under stress as well as during normal metabolic activity (Wasternack and Feussner, 2018). The resulting “oxylipins” show great variability depending on the carbon affected. Reactive carbonyls formed necessitate plant defense mechanisms. Mano et al. describe how aliphatic acrolein-type molecules and hydroxynonenals are detoxified by tau-class GSTs (GSTU). This metabolic activity applies to approximately 30% of GSTUs tested in *Arabidopsis*, distinguishing them as remarkable natural GST substrates. Regarding xenobiotic metabolism, Tzafestas et al. studied detoxification of trinitrotoluene (TNT) by *Arabidopsis* GSTs. The authors focused on the unusual finding that, between two GSTUs with 79% sequence identity, only one catalyzes substitution of a nitro group with reduced glutathione (GSH). The authors concluded this reaction and subsequent degradation may render the aromatic moiety more susceptible to cleavage, thus stimulating removal of TNT from the environment.

Regarding gene regulation, Baek et al. investigated expression of *GSTs* and other genes involved in detoxification and signaling in sorghum shoots to comprehensively understand tissue-specific expression following safener treatment (Riechers and Green, 2017). Interestingly, transcriptome analysis revealed strong induction of genes encoding several detoxification enzymes, including cytochrome P450s, GSTs, and glucosyl-transferases, and several upregulated GSTs were similar to enzymes involved with recycling the cyanogenic glycoside dhurrin. Additionally, a genome-wide association study identified two phi-class *GSTs* (*SbGSTF1/F2*) strongly associated with tolerance to the herbicide *S*-metolachlor. This information establishes a new framework for further studies on detoxification and signaling mechanisms for crop protection. Gallé et al. reviewed literature regarding effects of light quality, intensity, duration, and circadian rhythms on plant *GST*s. Patterns and regulation of *GST* expression were discussed in the context of diurnal variations in cellular GSH and reactive oxygen species levels. Importantly, light-regulated expression of GST enzymes possessing detoxification activities could affect whole-plant tolerance levels to abiotic or biotic stresses.

Numerous studies have shown that GSTs are involved in biotic stress responses. Gullner et al. proposed a model describing diverse roles of plant GSTs in interactions of plant hosts with pathogenic microbes considering four scenarios: (i) symptomless resistance, (ii) hypersensitive response-associated resistance, (iii) limiting susceptibility to systemic pathogen spread and plant cell/tissue death, and (iv) promoting susceptibility to biotrophic fungi and viruses. The authors’ concluded the most important function of GSTs in influencing plant-pathogen interactions is likely suppression of oxidative stress in infected host tissues. Upon pathogen recognition, secondary compounds (e.g., glucosinolates and indole-type phytoalexins) are induced in Brassicaceae species. Czerniawski and Bednarek summarized current knowledge on GST involvement in sulfur-containing secondary metabolites. Only AtGSTF6 and AtGSTU13 were required for their biosynthesis, but the roles of several other GSTs were suggested. One main conclusion is that specificities of these GSTs may result from their varying expression patterns and cellular/subcellular localizations.

GSTs may also have novel uses for biotechnology applications toward plant improvement (Perperopoulou et al., 2018). Chronopulou et al. employed a strategy to produce synthetic GSTUs by generating a cDNA library of GSTUs from abiotic stress-treated common bean (*Phaseolus vulgaris*) and soybean (*Glycine max*) using degenerate GST-specific primers and reverse transcription-PCR. This library was then diversified by directed evolution *via* a procedure called “DNA shuffling”. Using this method, the authors demonstrated the power of forced evolution for generation of variants (synthetic enzymes) with enhanced enzymatic properties that could be valuable in biotechnology. Stavridou et al. used transplastomic (i.e., plants whose transgene has been inserted into the chloroplast genome) tobacco lines as an alternative approach to nuclear transgene expression. Analysis of such lines expressing either of two different GSTs—an *Arabidopsis* theta-class GST normally expressed in the peroxisomes and a chimera engineered from two maize GSTUs—showed an increase in salt, osmotic, and oxidative stress tolerance. This information is of great importance for better understanding the role of GSTs in abiotic stress responses and development of stress-tolerant plants *via* plastome engineering. Dixon and Edwards utilized a protein-ligand fishing strategy to identify natural ligands for AtGSTU19 and AtGSTF2 expressed as *Strep*-tagged fusion proteins *in planta*. Following transient and stable expression in *Nicotiana* and *Arabidopsis*, respectively, the GSTs were recovered using *Strep*-Tactin affinity chromatography and bound ligands characterized by LC-MS. AtGSTF2 predominantly bound phenolic derivatives, whereas AtGSTU19 captured mainly glutathionylated oxylipin conjugates. Such ligand fishing has great potential for providing new insights into protein function *in planta* as well as identifying novel classes of natural product-derived enzyme inhibitors.

Sylvestre-Gonon et al. reviewed the serinyl-GST (Ser-GST) protein family, which have a conserved serine in their N-terminal active site. Ser-GSTs catalyze GSH conjugation reactions and display high peroxidase activity, both of which are important for stress tolerance and herbicide detoxification. Furthermore, Ser-GSTs participate in binding and transport of small heterocyclic ligands (e.g., flavonoids such as anthocyanins and polyphenols) through noncatalytic or “ligandin” properties. The authors discussed the known enzymatic and structural properties of Ser-GSTs and described their biochemical and physiological functions.

The current Frontiers research topic sheds new light on myriad functions of plant GSTs and provides an up-to-date, comprehensive understanding of the GST protein family by defining roles of great importance to endogenous plant metabolism, xenobiotic detoxification mechanisms, and tolerance to abiotic and biotic stresses. Several important questions remained unresolved and significant challenges need to be addressed in the future, however, to allow even deeper mechanistic insights into GST functions *in planta*. Critical knowledge gaps include identifying distinct structural and biochemical features of each subclass within the plant GST protein superfamily, molecules transformed and/or transported by GSTs* via *ligandin properties, molecular-genetic mechanisms and cellular factors that regulate precise cell- and tissue-specific expression of plant *GST* genes before and after stress, and exploring new proteins and the plant defense signaling pathways with which they interact. By highlighting the most recent discoveries in this exciting field of biology, we hope to stimulate further research into unravelling the complex roles of GSTs in plant physiology and crop improvement.

## Author Contributions

All authors listed made a substantial, direct, and intellectual contribution to the work and approved it for publication.

## Conflict of Interest

The authors declare that the research was conducted in the absence of any commercial or financial relationships that could be construed as a potential conflict of interest.
